# Search for potential reading frameshifts in cds from *Arabidopsis thaliana* and other genomes

**DOI:** 10.1093/dnares/dsy046

**Published:** 2019-02-04

**Authors:** Y M Suvorova, M A Korotkova, K G Skryabin, E V Korotkov

**Affiliations:** 1 Institute of Bioengineering, Research Center of Biotechnology of the Russian Academy of Sciences, Moscow, Russia; 2 National Research Nuclear University MEPhI (Moscow Engineering Physics Institute), Moscow, Russia

**Keywords:** reading frameshift, periodicity, dynamic programming, genetic algorithm

## Abstract

A new mathematical method for potential reading frameshift detection in protein-coding sequences (cds) was developed. The algorithm is adjusted to the triplet periodicity of each analysed sequence using dynamic programming and a genetic algorithm. This does not require any preliminary training. Using the developed method, cds from the *Arabidopsis thaliana* genome were analysed. In total, the algorithm found 9,930 sequences containing one or more potential reading frameshift(s). This is ∼21% of all analysed sequences of the genome. The Type I and Type II error rates were estimated as 11% and 30%, respectively. Similar results were obtained for the genomes of *Caenorhabditis elegans,* Drosophila *melanogaster, Homo sapiens, Rattus norvegicus* and *Xenopus tropicalis*. Also, the developed algorithm was tested on 17 bacterial genomes. We compared our results with the previously obtained data on the search for potential reading frameshifts in these genomes. This study discussed the possibility that the reading frameshift seems like a relatively frequently encountered mutation; and this mutation could participate in the creation of new genes and proteins.

## 1. Introduction

The occurrence of reading frameshifts in a gene is a very serious mutation, and it results in the creation of mutant proteins.^1^ This may result in various hereditary^2–4^ or oncological diseases.^5^ The occurrence of reading frameshifts in a gene is caused by the insertion or deletion of nucleotides, which is not a multiple of 3. Besides, in eukaryotic genes, reading frameshifts could arise through the shift of boundaries between exons and introns, when the splicing point is under mutation.^6^ In the evolution of genetic sequences, it is common to observe the insertions and deletions of small fragments.^7^^,^^8^ In this case, within the translation stage there is a complete change in the amino acid sequence beyond the frameshift position.^9^ Consequently, the encoded amino acid sequence can completely lose its biological function and become a pseudogenе. This sequence could either acquire another biological function or its function may remain the same. The study of such mutations is important for understanding the mechanisms of protein sequence evolution.

Errors of genome sequencing and assembly processes could also cause reading frameshifts in genes present in modern databases.^10^ For example, in pyrosequencing methods and PacBio sequencing technology, frameshift errors appear very frequently. In this case, the encoded amino acid sequence beyond the frameshift position would be incorrect, which makes further annotation difficult. To find and correct shifts for these sequencing methods, special programs have been developed.^11^^,^^12^ The identification of such errors is important for improving annotation and ensuring further research of new genomes.

In the literature, cases of programmed ribosomal frameshifts have been reported. In this case, the ribosome itself shifts within the protein synthesis process on a single base, which results in the emergence of an alternative protein.^13^ However, such events are beyond the scope of this study. The present study is focused on developing a mathematical algorithm for the detection of potential reading frameshifts in protein-coding sequences (cds).

The methods currently used to find reading frameshifts can be divided into two classes. The methods of the first class are based on the comparison (alignment) of sequences. These methods use protein database search to find sequences which are homologous to the sequence of interest but are devoid of the frameshift. Such methods suggest the use of the BLAST program and analogues.^8^^,^^9^^,^^14^ An obvious limitation of these methods is that in the absence of a homologous sequence without a frameshift, it is impossible to determine the presence of a frameshift. The large number of substitutions that occurred after the frameshift event could also make it difficult to find similarities. In order to search for remote similarities, while taking the possible frameshifts into account, special programs are being developed.^15^

The methods of the second class are aimed at finding the frameshift directly by the sequence (*ab initio*).^16–18^ There are several methods for the prediction of protein-coding regions in genome sequences that consider the probable reading frameshifts. These methods are used by programs: FrameD,^16^^,^^19^ based on the Markov models, program for predicting genes by taking into account frameshifts and searching for frameshifts in known genes. The FragGeneScan program^20^ was created to search for coding regions in short reads taking into account the frameshifts and based on the Hidden Markov Models (HMMs). Also, the HMM-frame program^12^ uses the HMMs to search for protein domains in the metagenomics sequences while taking into consideration the probable reading frameshifts.

The GeneTack program is among the most widely used tools.^17^^,^^21^ This program can identify reading frameshifts resulting from mutations and sequencing errors. The idea of the algorithm is that several genes are located on the chromosome one after another and are represented in databases as independent genes which may have originated from a single coding sequence separated as a result of the reading frameshift. The GeneTack algorithm is based on the HMM and the Viterbi algorithm, and possesses several parameters that require adjustment prior to sequence processing. Unfortunately, the methods are all limited because they require a training sample for determination of the HMM parameters. In the result some statistical properties of the gene could be averaged, which significantly reduces the capability of the methods. In particular, the frequencies of *k*-words belong to these statistical properties. Training is carried out using another program of the authors—GeneMarkS.^22^ The training sample is usually the entire sequence of the genome under study (for prokaryotes) or a set of coding sequences of this genome (for eukaryotes). Besides, a prepared model from the authors’ website, having a similar level of GC content, could also be used. The GC content of the sequences under examination has a significant effect on the search for reading frameshifts. The given method was designed to search for reading frameshifts both at the genome annotation stage (as part of the genome annotation integrated programs) and in known sequences.

**Figure 1:**
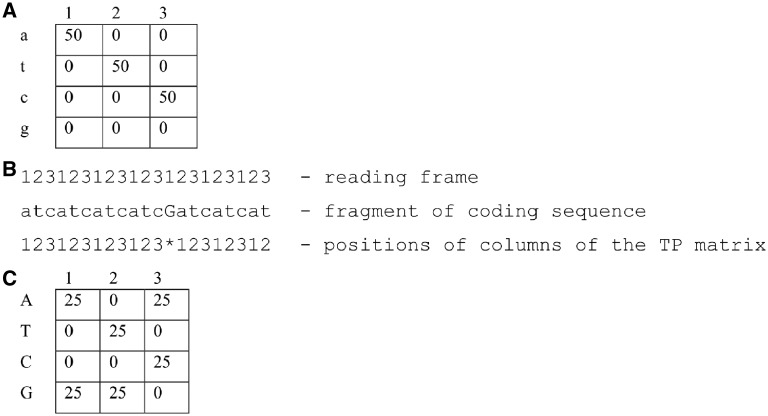
(A) The TP matrix is shown for the sequence *S*={*atc*}_50_. (B) The phase TP in a fragment of the sequence S. A base *g* is inserted in the middle of the sequence. The phase *Fa *=* *0 was before the insertion of *g*, because there is an agreement between the columns of the matrix and the positions of the codons as 1 => 1, 2 => 2, 3 => 3, respectively. *Fa *=* *1 after the insertion of *g*, because the following agreement is observed: 1 => 2, 2 => 3, 3 => 1. (C) The TP matrix for the sequence *S*={atcgga}_25_.

Most of the methods used for the detection of frameshifts *ab initio* are based on the well-known property of the cds, i.e. the triplet periodicity (TP). TP is exclusively present in the cds of virtually all organisms and is considered to be a consequence of the preferred use of synonymous codons by various organisms.^23^^,^^24^ This property is used by many programs designed for cds prediction.^25^^,^^26^ It has been shown that the reading frameshift in a gene resulted in TP phase shift in the corresponding position.^18^ TP phase shift is the shift of TP matrix columns relative to the positions of the bases in the codons. An example is presented in Fig. 1A and B. Figure 1A shows the TP matrix, which was constructed from the coding sequence ‘atcatcatc …’. The first column of this matrix corresponds to the first codon base, the second column corresponds to the second codon base, and the third column corresponds to the third codon base. Conditionally, this can be shown as: 1 => 1, 2 => 2, 3 => 3. Such a relationship can be called Phase 0, or *Fa *=* *0. After inserting one base (G in the middle of the sequence, Fig. 1B), the TP phase shifts to one base. In this case, the correspondence of positions in the codons and columns of the matrix is 1 => 2, 2 => 3, 3 => 1. This correspondence is called Phase 1 or *Fa *=* *1. The value *Fa *=* *2 is also possible. In this case, 1 => 3, 2 => 1, 3 => 2. Such a correspondence will be observed when inserting any two DNA bases. After inserting *n* DNA bases, *Fa* becomes *n* − 3int(*n*/3). Here, ‘int’ is the operation of computing the integer part of a number.

For the determination of TP phase shifts, mathematical methods such as the Fourier transform,^27^ wavelet transform,^28^ dynamic programming^29^ or methods based on comparison of periodicity matrices^30^ have been employed. The Fourier transform method produced good results on artificial sequences and genes with a high level of TP. However, the method requires a window of rather long length (the authors recommended a window size of about 750 nt).^27^ Methods based on dynamic programming or matrices comparison are characterized by enhanced sensitivity, but are incapable of detecting frameshifts in sequences with a low level of TP.^18^

We developed a new approach for TP phase shift detection in cds from prokaryotic and eukaryotic genomes. By this method, an attempt was made to eliminate the disadvantages of the HMM approaches connected to the requirement of the HMM configuration in the sampling of genes. Such adjustment significantly averages all the characteristics of the genes TP, because different classes of TP exist in the genome, and the combination thereof decreases the statistical significance of TP.^31^ In addition, the use of HMM could identify reading frameshifts only in those cds possessing the same base correlations, as the training sample. If the correlations between bases of cds are different, then it could be impossible to detect the frameshifts. The HMM limitations are considered in more details in the ‘3.4. Comparison with the Genetack-GM program’ section.

We developed a method that enables determination of the best TP matrix for each sequence while taking into account the correlation of the adjacent DNA bases along with the possibility of nucleotides insertion or deletion. The genetic algorithm and the dynamic programming method^32^ were used in a similar way, as described in our previous work on amino acid sequences. However, in the given case, the algorithm was modified by introducing a matrix that considers the correlation of neighbouring bases. After identifying such a matrix, we performed the final alignment of the sequence of interest with respect to the best matrix and found the probable reading frameshift positions.

As a result of our research, we identified an unexpectedly large number of probable reading frameshifts in the *Arabidopsisthaliana* genome cds, which constituted 9,930 with the error level of the first and second types being ∼11% and 30%. This is about 21% of all the registered cds from this genome. We obtained similar results for the genomes of Caenorhabditis *elegans,* Drosophila *melanogaster, Homo sapiens,* Rattus *norvegicus* and *Xenopus tropicalis*. It was assumed that the reading frameshift is a relatively frequently found mutation, and this mutation could take part in the creation of new genes and proteins.

## 2. Mathematical methods and algorithms

### 2.1. General description of the mathematical algorithm used in this work

The task of reading frameshift detection in a protein-coding sequence could be mathematically solved, if it becomes possible to relate the statistical properties of the sequence with the reading frame. In this case, one could find the reading frameshift in a sequence without using any kind of training and without involving any additional information. Such a property could be presented by the TP of genes.^33^ In this case, the reading frameshift would appear as a TP phase shift.

Let us consider a protein-coding sequence *S* having a length *N.* The TP of a sequence is usually set in the form of the *MT*(3, 4) matrix. Here, the columns indicate the three positions of codons (1, 2 or 3), and the rows represent four types of nucleotides.^33^Figure 1A and C presents an example of a TP matrix created for the sequences *S*={atc}_50_ and *S*={atcgga}_25_. In the first case, only three matrix elements are filled, whereas six elements are filled in the second case. The TP of the *S* sequence set in the form of the *MT* matrix perfectly reflects the difference in base frequencies at each position of the codon from the base frequencies throughout the entire nucleotide sequence. However, the *MT* matrix does not consider the correlation between adjacent bases, as illustrated by the following example. Let us assume that in our sequence only four codons are equally likely to be used: ATA, TAT, CGC and GCG, and they are arranged in a sequence in some random order. Then, the DNA base frequencies in each column of the *MT* matrix would be equal to the frequencies of bases in the entire sequence, and the matrix constructed according to this sequence would show that there is no TP in the sequence.

Therefore, it is more appropriate to use another matrix *M*(*i, n*) while searching for TP. The matrix contains 16 rows and 3 columns. The columns of the matrix represent the pairs of positions in the codon: 3-1, 1-2 and 2-3. Here, the *i* column of the *M* matrix takes the 1, 2 and 3 values for pairs of positions in codon: 3-1, 1-2 and 2-3, respectively. This means that the *M* matrix columns are numbered by the last position out of the two. The *n* row number shows the frequency of the base pairs in columns, and *n* takes values from 1 to 16. In order to fill the *M* matrix, the row number is calculated as follows: *n *=* s*(*j* − *1*)+4(*s*(*j*) − 1). This study utilized the *a *=* *1, *t *=* *2, *c *=* *3 and *g *=* *4 nucleotides numerical coding. Here, *s*(*j*) is the base of the *S* sequence in the *j* position, which corresponds to the *i* position in the codon calculated with the following equation: *i *=* j* − 3int(*j*/3), where ‘int’ is the integer part of the number. Then, the *s*(*j* − 1) base corresponds to the previous neighbouring codon position. Thus, *j* ranges from 2 to *N* values. For each *j*, *M*(*i, n*) = *M*(*i, n*) + 1.Therefore, the *M*(3, 16) matrix contains two types of statistical regularities of the coding regions. The first one is the difference between the frequencies of nucleotides in each position of a codon and the nucleotide frequencies of the entire *S* sequence. The second one is the correlation of two neighbouring nucleotides positions of the codon (3-1, 1-2 and 2-3).

Assuming we have a sequence *S* that is a cds. The sequence *S*_0_ is sequence *S* prior to the reading frameshifts, that is, *S*_0_ is the ancestor of sequence *S*. We create the matrix *M*(3, 16) for *S* and we create the matrix *M*_0_(3, 16) for *S*_0_ as described above. If one knows the *M*_0_(3, 16), then such TP periodicity shift could be found using the alignment of sequence *S* with the position-weight matrix (PWM) *W*_0_ (see Equation 1). This matrix was created using *M*_0_(3, 16), as it was performed previously for the *MT*(3, 4) matrix^34^ and is shown below in Equation (1). To create the *W*_0_(3, 16) matrix, each element of the *M*_0_(3, 16) matrix was transformed to the argument of the normal distribution, using the normal approximation for the binomial distribution. Dynamic programming could be used to find the global alignment of the *S* sequence with respect to the *W*_0_(3, 16) matrix. This alignment can be performed by cyclic alignment, as was done previously.^35^ However, the problem is that there are no stored *M*_0_(3, 16) matrix and the corresponding *W*_0_(3, 16) matrix for the sequence *S*_0_ and sequence *S* is unknown. Using the sequence of an existing gene, it is impossible to identify *M*_0_(3, 16) and *W*_0_(3, 16) matrices. The reason why it is impossible to calculate this matrix is the uncertainty of the reading frameshift positions in the gene.

For example, let us consider a sequence *S*_0_:{atg}_50._ If we delete a single nucleotide in the middle of *S*_0_, we obtain a sequence *S*={atg}_25_{tga}_25_. The matrices constructed for the sequences *S*_0_ and *S* [*M*_0_(3, 16) and *M*(3, 16)] are shown in Table 1A and B, respectively. Table 1B shows that for the *S* sequence, all available base pairs that could be constructed from the existing reading frame would receive a similar positive weight, when the *M*(3, 16) matrix is transformed into the *W*(3, 16) matrix (PWM). Thus, using the global alignment of the *S* sequence with respect to the *W*(3, 16) matrix^36^ (or of the HMM^37^), it would be impossible to find the TP periodicity shift. This is because the deletion in position 76 of the *S* sequence shall not be created by global alignment with the *W*(3, 16) matrix. Such a shift could only be found if the *M*_0_(3, 16) matrix is known, and the *W*_0_ matrix is created on its base. Therefore, the task is to develop a mathematical method to find the *W*_0_ matrix and perform a global alignment of the *S* sequence relative to *W*_0_.

**Table 1:** (A) Matrixes *M*_0_(3, 16) and (B) M(3,16)

(A)					(B)				
	*N*	1	2	3		*N*	1	2	3
aa	1	0	0	0	aa	1	0	0	0
ta	2	0	0	0	ta	2	0	0	0
ca	3	0	0	0	ca	3	0	0	0
ga	4	50	0	0	ga	4	25	0	25
at	5	0	50	0	at	5	25	25	0
tt	6	0	0	0	tt	6	0	0	0
ct	7	0	0	0	ct	7	0	0	0
gt	8	0	0	0	gt	8	0	0	0
ac	9	0	0	0	ac	9	0	0	0
tc	10	0	0	0	tc	10	0	0	0
cc	11	0	0	0	cc	11	0	0	0
gc	12	0	0	0	gc	12	0	0	0
ag	13	0	0	0	ag	13	0	0	0
tg	14	0	0	50	tg	14	0	25	25
cg	15	0	0	0	cg	15	0	0	0
gg	16	0	0	0	gg	16	0	0	0

**Figure 2:**
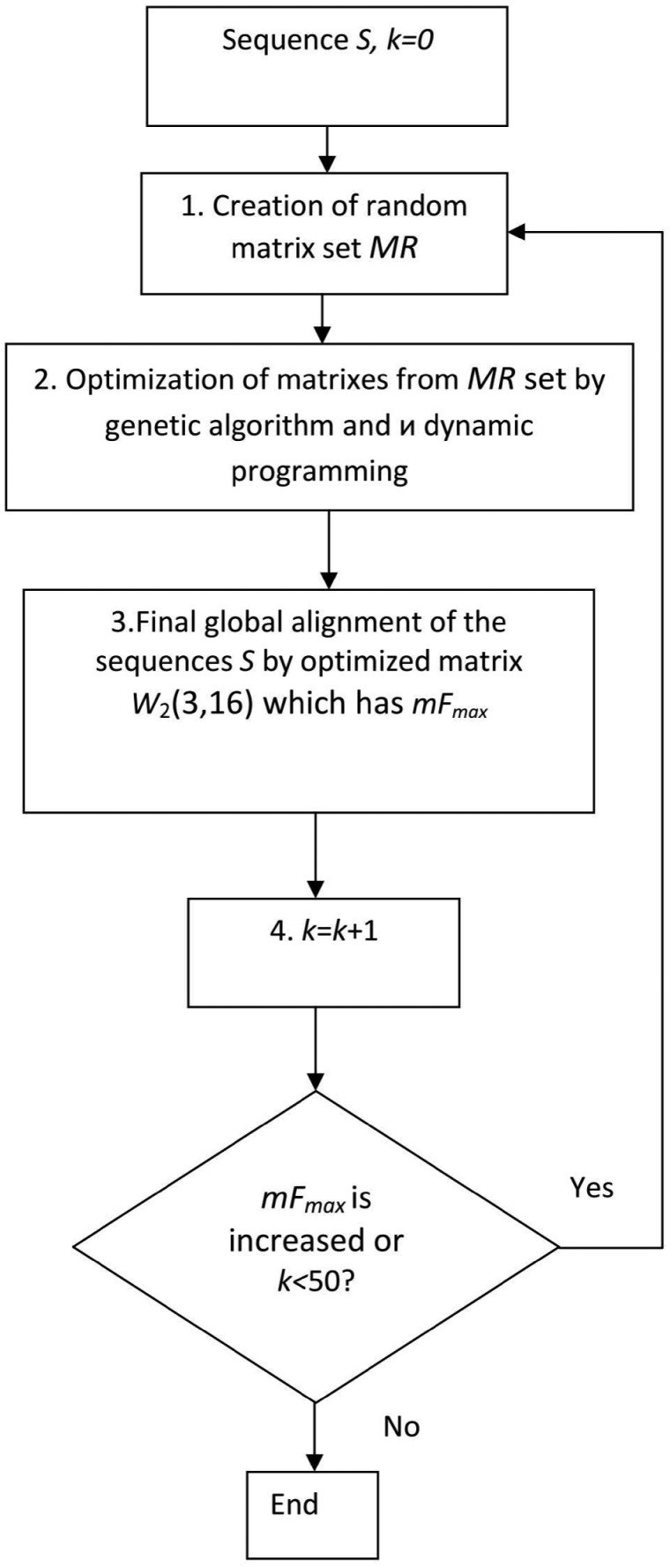
A block diagram of the algorithm for the optimization of random matrices M(3,16) from the *MR* set, used to search a matrix with the largest *mF*_max_.

**Figure 3:**
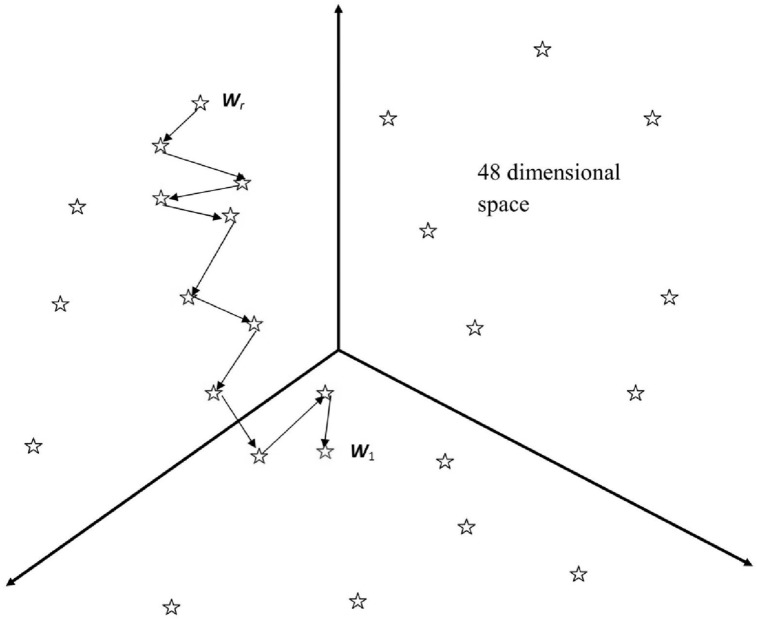
The idea of the algorithm is to create a random matrix *W*_r_, (or a set of random matrices), then optimize it with a genetic algorithm and get the *W*_1_ matrix as a result of optimization.

This study partially utilized the algorithm that we developed in our previous work.^32^ This algorithm employs a particular property of the *P*(*x *>* F*) probability, which is calculated for the global alignment of the *S* sequence with the PWM. It was shown that the value *P*(*x *>* F*) is lower for the *W*_0_(3, 16) matrix compared with the *W*(3, 16) matrix. Here, *F* is the similarity function value of the global alignment (see Equation 2). This means that we must elaborate on a procedure for optimizing the *W*(3, 16) matrix to find the *W*_1_(3, 16) matrix that is closest to the *W*_0_(3, 16) matrix. In this case, we obtained the maximum value of the *F* similarity function and the lowest value of the *P*(*x *>* F*) probability, as well as the global alignment of the *S* sequence with the *W*_1_(3, 16) matrix. This global alignment allows us to determine the coordinates of the potential reading frameshifts. The optimization was performed using the algorithm shown in Fig. 2. The idea of the algorithm is to create a random matrix *W*_r_(3, 16) (or a set of random matrices), followed by optimization with a genetic algorithm and obtaining the *W*_1_(3, 16) matrix as a result of optimization. Matrices have 3 columns and 16 rows, hence optimization takes place in a 48-dimensional space. Schematically, this optimization is presented in Fig. 3. At each optimization step, we move along the 48-dimensional space and obtain the intermediate matrices, which are represented by asterisks. Let us consider the crucial points of this algorithm.

### 2.2. Methods for creating a *W*_2_(3, 16) random matrix

Assuming we have a DNA sequence *S* (cds), to create a *W*_2_(3, 16) random matrix, we used the *S*_2_ sequence, which was obtained from the *S* sequence by randomly shuffling the nucleotides. The random shuffling algorithm has already been described in detail.^32^ To do this, a sequence of *RS* of the same length as the *S* sequence was generated by a random number generator. Thereafter, we sorted the sequence *RS* in ascending order and memorized the permutations. Thereafter, these permutations were performed in the sequence *S* and the sequence *S*_2_ was obtained*.* Then, using the *S*_2_ sequence, we filled the *M*_2_(3, 16) matrix. We filled the *W*_2_(3, 16) matrix according to the equation:

(1) $$W_{2}\left(i,k\right)=\frac{M_{2}\left(i,k\right)-(N-1)p_{2}(k)}{\sqrt{\left(N-1)p_{2}(k)(1-p_{2}(k)\right)}}$$

Here $p_{2}(k)=p(l)p(m)$, where *p*(*l*) and *p*(*m*) are the probabilities of the *l-* or *m-*type nucleotides in the *S_2_*sequence (*l, m*∈{a, t, c, g}); *p*(*l*) *= q*(*l*)*/N*, *q*(*l*) is the number of *l-*type nucleotides in the *S*_2_ sequence, and *N* is the *S*_2_ sequence length. This PWM calculation considers two types of statistical regularities in the *S*_2_ sequence. On the one hand, it considers heterogeneities in the nucleotide frequencies at each codon position, because the *p*_2_ probability without specificity to each codon position is used. On the other hand, this matrix also considers the correlation of neighbouring bases, because the expected number of each of the 16 pairs is calculated for each *i* position in the codon, *i*∈{1, 2, 3}.

### 2.3. Optimization of the *W*_2_(3, 16) matrix using genetic algorithm and dynamic programming

#### 2.3.1. Application of dynamic programming for *W*_2_(3, 16) matrix optimization

Assuming we have a DNA sequence *S*, then we optimized the corresponding *W*_2_(3, 16) matrix by a genetic algorithm to maximize the similarity function *F*. To calculate the similarity function, the *S* sequence was aligned with respect to the *W*_2_(3, 16) matrix by an iterative procedure, and the *F* value was calculated:

(2) $$\begin{array}{c} F(i,j)=\max\left\{\begin{array}{c} F(i-1,j-1)+W_{2}^{t}(a(i),n)\\ F(i,j-1)-d\\ F(i-1,j)-d \end{array}\right\}. \end{array}$$

Here, *i* runs from 1 to *N + h*, and *j* runs from 2 to *N*. The *h* constant indicates the maximum number of insertions and deletions that could appear in the final alignment. We choose it as equal to 50, because among the studied sequences there was no cds with the number of insertions or deletions >50. Here, *a*(*i*) was calculated as *i* − 3int(*i*/3.0). Therefore, *a*(*i*) takes the values 1, 2, 3, 1, 2, 3, 1, 2, 3, … for *i *=* *1, 2, 3, 4, 5, 6, 7, 8, 9, … . $W_{2}^{t}$ is the transformed *W*_2_ matrix. The transformation ($W_{2}$*=>*$W_{2}^{t}$) was carried out so that all matrices $W_{2}^{t}$ (which are used in Equation 2) would have the same value *R*^2^ = *R*_0_ and *K_d_* = *K*_0._ For any matrix $W_{2}^{t}$, the constants *R* and *K_d_*can be calculated using the following equations:

(3) $$R^{2}=\sum_{i=1}^{3}\sum_{j=1}^{16}w_{2}^{t}{\left(i,j\right)}^{2},$$

(4) $$K_{d}=\sum_{i=1}^{3}\sum_{k=1}^{16}w_{2}^{t}(i,k)p(i)p_{2}(k).$$

*p*(*i*) is the probability of encountering the symbols 1, 2 and 3 in the sequence *a*(*i*) and it is 1/3 for any *i*. The probability *p*_2_(*k*) is defined in Equation (1) above. For all calculations in this work, we used *R*_0_ = 1,050 and *K*_0_ = −1.8. These values were chosen based on the following considerations. *R*_0_ was calculated as the sum of squares of the matrix *W*_2_. The matrix *W*_2_ was created for the sequence *S* = {*atg*}_400_ with the introduction of 2.0 random substitutions per nucleotide. The sequence *S* had a level of TP, which is average for the genes under study. We estimated the level of TP based on the *MT* matrix using mutual information converted into an argument of normal distribution *X*. The average level of *X* for the analysed genes was about 6.2. These calculations have earlier been described in detail.^38^

The value *K*_0_ was chosen using a *Gr* set of artificially created sequences. The value of *K*_0_ reflects the accuracy of determining the beginning and the end of the local alignment and the number of values which are greater or less than zero in the matrix $W_{2}^{t}$.^3^^2^ The volume of the set *Gr* was 500 sequences. Each sequence had a length of 1,200 nt. The first 400 bases of this sequence were random. The next 400 bases correspond to some random matrix *MT* for which the TP level *X *=* *6.2 (see the previous section). Then again, follow a random sequence of 400 bases. The matrix *MT* was used to calculate the weight matrix *WT* according to the equation:

(5) $$WT\left(i,j\right)=\frac{MT\left(i,j\right)-Lp(i,j)}{\sqrt{Lp(i,j)(1-p(i,j))}}.$$

Here, *p*(*i*, *j*) = *x*(*i*)*y*(*j*)/*L*^2^, $x(i)=\sum_{j=1}^{4}MT(i,j)$, $y(j)=\sum_{i=1}^{3}MT(i,j)$ and $L=\sum_{i=1}^{3}\sum_{j=1}^{4}MT(i,j)$. Then, for each *WT* matrix, we constructed a local alignment according to Equation (6). Here we use the sequences *a*(*i*) also, the indices *i* and *j* are the same as in Equation (2).

(6) $$\begin{array}{c} E(i,j)=\max\left\{\begin{array}{c} 0\\ E(i-1,j-1)+WT(a(i),j)\\ E(i,j-1)-d\\ E(i-1,j)-d \end{array}\right\}. \end{array}$$

We found *E*_max_, the coordinates of the maximum *i*_max_ and *j*_max_ as well as the coordinates of the beginning of the alignment *i*_0_ and *j*_0,_ for each sequence from the set *Gr*. We tested different values of *K*_0_ and chose *K*_0_ = −1.8. In this case, the sum of differences *i*_0_ and *j*_0_ from 400, plus the sum of the differences *i*_max_ and *j*_max_ from 800 were minimal and equal to 46 nt.

Let us continue to consider Equation (2). For this equation *n *=* s*(*k*) + 4(*s*(*j*) − 1) ranges from 1 to 16. The index *k* is calculated using transitions already created in the *F* matrix. As the matrix $W_{2}^{t}(a(i),n)$ is calculated for pairs of bases, to calculate *k*, it is necessary to determine the previous base of sequence *S*, which is included in the alignment. The previous base can be found by calculating the path to the cell with coordinates (*i*, *j*). If we get to the (*i, j*) cell from the (*i* − 1*, j* − 1) cell and we get to the (*i* – 1*, j* – 1) cell from the (*i* – 2*, j* – 2) cell, then *k *=* j* – 1. This corresponds to the transitions *F*(*i* – 2, *j* – 2) => *F*(*i* – 1, *j* – 1) => *F*(*i*, *j*) in the matrix *F*. Such a move corresponds to the diagonal move, and there are no insertions or deletions. If we get to the (*i*, *j*) cell from the (*i* − 1, *j* − 1) cell, to the (*i* − 1, *j* − 1) cell from the (*i* − 1, *j* − 2) cell and to the (*i* − 1, *j* − 2) cell from the (*i* − 2, *j* − 3) cell, then *k *=* j* − 2. This move corresponds to the skipping of a single base in the sequence *S*. Therefore, the transitions in the matrix *F* are *F*(*i* − 2, *j* − 3) => *F*(*i* − 1, *j* − 2) => *F*(*i* − 1, *j* − 1) => *F*(*i*, *j*). The longer deletions in the sequence *S* (not longer than *h*) are treated similarly. Assuming that there is a deletion of length *q* in the sequence *S*; this means that *k *=* j* − 1 − *q* and the path to the cell (*i*, *j*) is *F*(*i* − 2, *j* − 2 − *q*) => *F*(*i* − 1, *j* − 1 − *q*) => … => *F*(*i* − 1, *j* − 2) => *F*(*i* − 1, *j* − 1) => *F*(*i*, *j*).

Deletions can also occur in the sequence *a*(*i*). These deletions correspond to a skipping of columns of the matrix $W_{2}^{t}(a(i),n)$in the resulting alignment. The path *F*(*i* − 3, *j* − 2) => *F*(*i* − 2, *j* − 1) => *F*(*i* − 1, *j* − 1) => *F*(*i*, *j*) corresponds to a skipping of a single column of the $W_{2}^{t}$ matrix. In this case, we cannot use the matrix $W_{2}^{t}$ because it uses pairs of neighbouring bases. Therefore we need a weight matrix calculated for pairs of bases separated by one base, i.e. the pairs *a*(*i*)*a*(*i *+* *2), *i *=* *1, 2, …, *N *+* h* − 2 [*N *+* h* is the length of the sequence *a*(*i*)]. Therefore, we need another matrix ($W_{3}^{t}$) which could be obtained from the $W_{2}^{t}$ matrix. The $W_{3}^{t}$ matrix contains weights for pairs of bases which are in codon positions 2-1, 3-2 and 1-3 (i.e. separated by one codon position).

(7) $$W_{3}^{t}(x,i+4(l-1))=0.25\sum_{j=1}^{4}(W_{2}^{t}(x,i+4(j-1))+W_{2}^{t}(x,j+4(l-1)))/2.0.$$

*i*, *j* and *l* denote the bases: 1 = *a*, 2 = *t*, 3 = *c*, 4 = *g*. Equation (7) is based on the assumption that a weight of the pair of bases (*i*)(*l*) separated by the single base *j* can be calculated using the weights of two intersecting pairs of bases (*i*)(*j*) and (*j*)(*l*)*.* Therefore, one can use the averaged weight of four possible intersecting pairs to estimate the weight of the pair (*i*)(*l*). Here *i* and *l* are fixed, and *j* ranges from 1 to 4.

A deletion of two columns of the matrix $W_{2}^{t}$ corresponds to the path *F*(*i* − 4, *j* − 2) => *F*(*i* − 3, *j* − 1) => *F*(*i* − 2, *j* − 1) => *F*(*i* − 1, *j* − 1) => *F*(*i*, *j*). In this case, the equation for the calculation of the matrix$W_{3}^{t}$ is as follows:

(8) $$W_{3}^{t}(x,i+4(l-1))=0.0625\sum_{j=1}^{4}\sum_{k=1}^{4}(W_{2}^{t}(x,i+4(j-1))+W_{2}^{t}(x,k+4(l-1)))/2.0.$$

Here *i*, *j*, *k* and *l* also denote the bases: 1 = *a*, 2 = *t*, 3 = *c*, 4 = *g*. Equation (8) is based on the assumption that the weight of a pair of bases (*i*)(*l*) separated by two bases (*j* and *k*) can be calculated based on the weights of the neighbouring pairs (*i*)(*j*) and (*k*)(*l*). Therefore, to estimate the weight of the pair separated by two positions, one can use the average weight of 16 possible neighbouring pairs. Here *i* and *l* are fixed, and *j* and *k* run from 1 to 4. If we delete three columns of the matrix, we return to the matrix$W_{2}^{t}$. This will be correct for all deletions that are multiples of 3. Therefore, we used Equation (7) for column deletions of length 1, 4, 7, … and we used Equation (8) for column deletions of length 2, 5, 8, ….

The zero row and column of the *F* matrix were filled with negative numbers, and the *F*(0, 0), *F*(1, 0), …, *F*(*h*, 0) were 0. For transition from the zero column to the first column of the *F* matrix and from the zero row to the first row of the *F* matrix, the $W_{4}^{'}$*F* matrix was used. It was determined as follows:

(9) $$W_{4}^{\text{t}}(x,n)=0.25\sum_{i=1,4}W_{2}^{\text{t}}(x,i+4(n-\text{1})).$$

Here, averaging over the four previous bases occurs and the weight depends only on the base in position *j*. In this case, the $W_{4}^{'}$ matrix replaces the $W_{2}^{t}$ matrix, but *n *=* s*(*j*) according to Equation (2).

**Figure 4:**
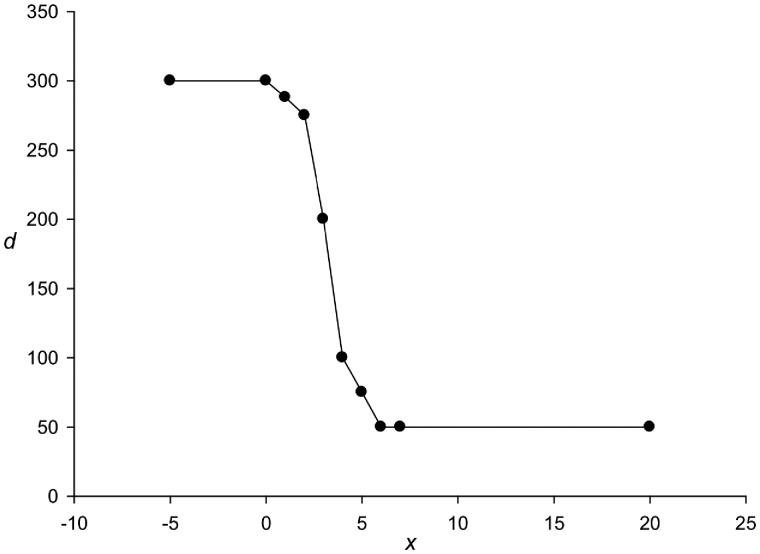
The dependence of the *d* value (see Equation 2) on the TP, of the analysed sequence. The TP was calculated using the TP matrix^33^ and is expressed in the arguments of the normal distribution and is shown along the *x*-axis.

The *d* constant (see Equation 2) plays an important role. It is intuitively clear that the smaller the statistical significance of TP in the *S* sequence, the higher the *d* value should be. To select the *d* value, we generated 1,000 sequences of 600 nt long for each level of the TP in the form of the *x* normal distribution argument^33^ in the interval from 0 to 20 and with a step of 1. Then in a random position of this sequence, no closer than 100 nt from the beginning and end, a deletion of one base was introduced. Let us call each set of such sequences *MP*(*x*). For each *MP*(*x*) set, we selected the *d* value in such a way that the number of insertions or deletions that were made by the method outside the distance (±50) from the artificial deletion did not exceed 5%. The obtained *d* values are presented in Fig. 4.

Simultaneously with the *F* matrix, the inverse transition matrix was also filled, as is usually the case when searching for global alignment. Then, using the inverse transition matrix, the alignment of the *S* sequence with respect to the $W_{2}^{t}$ matrix was constructed, and the value of the *F*_max_ similarity function in the cells of the matrix *F*(*N*, *N*)*, F*(*N *+* *1, *N*), …, *F*(*N *+* h*, *N*) was determined.

The transformation of matrix *W*_2_ into matrix $W_{2}^{t}$ makes possible the achievement of similar distributions for the values of *F*_max_ on the set of random sequences of *S* for different *W*_2_ matrices. This is the essence of the transformation of matrix *W*_2_ into matrix $W_{2}^{t}$. The similarity of distributions enables us to use *F*_max_ as a measure of the similarity of the matrix $W_{2}^{t}$ and the analysed sequence *S*. This allows consideration of the $W_{2}^{t}$ matrix having the largest value of *F*_max_ as the matrix that best represents periodicity in sequence *S*. It is possible to carry out all the calculations without such transformation and using $W_{2}^{t}$. Also, it is possible to use matrix *W*_2_ instead of matrix $W_{2}^{t}$ in Equation (2). However, the comparison of *F*_max_ for different *W*_2_ matrices will have to be done by the Monte Carlo method, which considerably slows down implementation of the genetic algorithm (Section 2.3.2).

#### 2.3.2. Application of the genetic algorithm for *W*_2_(3, 16) matrix optimization

When implementing the genetic algorithm, we considered the *F*_max_ value as the target function whereas the *W*_2_(3, 16) matrix was considered as the ‘organism’. The use of the genetic algorithm for optimizing the *W*_2_(3, 16) matrix was examined in detail in Ref. 32, and the reader is hereby referred to that publication. Let us consider in general the operating process of the genetic algorithm. First, the *MR* set of the *W*_2_(3, 16) random matrices with a volume of 500 matrices was generated. Each matrix was created as described in Section 2.1. Thereafter, each matrix was transformed to obtain a set of matrices with the same *R*^2^ and *K_d_*, as described in Section 2.3.1 and in Ref. 32. Consequently, a number of *MR*^t^ matrices were obtained. Each matrix from the *MR*^t^ set was subjected to the dynamic programming procedure to align with the *S* sequence, and the *F*_max_ value was calculated as described in Section 2.3.1. *F*_max_ was considered as the target function. Thereafter, two matrices from the *MR*^t^ set were selected, and the higher the probability of selecting these matrices, the greater was the value of the *F*_max_ objective function thereof. These two matrices ‘intercrossed’, and a ‘descendant’ was created. A descendant is a matrix, which possesses part of the cells from one matrix and a part of the cells from another. Then, one matrix from the *MR*^t^ set ‘perished’, and the probability of collapse was greater, the lower the *F*_max_ value for this matrix, and its place was taken by the descendant. In addition, random ‘point’ mutations were introduced in 10% of the randomly selected matrices from the *MR*^t^ set. Also, the greater the probability that the chosen matrix would be selected to introduce random mutations, the lower was its *F*_max_. Mutations were introduced into a random cell, and the number contained there was changed to a random number in a uniform interval from −5 to +5. Let us call the entire process an iteration. Therefore, within a single iteration, the *F*_max_ value is calculated in 500 matrices, one matrix is deleted, one descendant is created, and random ‘point’ mutations are introduced into 50 matrices. Let us call *mF*_max_ the maximum value for *F*_max_, which is obtained within a single iteration of the *MR* set. Then, the *MR* set is replaced by the *MR*^t^ set and the process is repeated from the very beginning.

In the result of the genetic algorithm operation, the *mF*_max_ was continuously increased. The genetic algorithm operation was stopped after the *mF*_max_ value stopped increasing during 50 iterations. On average, about 9 × 10^3^ iterations are required to reach this point.

### 2.4. Developing a measure of significance for the phase shifts of TP

**Figure 5:**
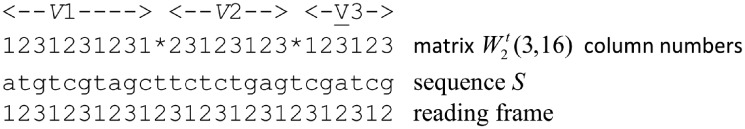
Scheme of division of *mF*_max_ on *V*1, *V*2 and *V*3 (see Section 2.4).

After carrying out the genetic algorithm, we obtained a single *W*_1_(3, 16) matrix (Section 2.1) which possesses the maximum similarity function (*mF*_max_) and the alignment of the *S* sequence with respect to the columns of the *W*_1_(3, 16) matrix. We are not interested in the *mF*_max_ values itself (it characterizes the TP level in the sequence), but rather in the statistical significance of the found TP phase shifts. To estimate it, the *mF*_max_ value was divided into three parts using the alignment sequence *S* and the *W*_1_(3, 16) matrix. The first part is the region of the *S* sequence, where the positions of the *W*_1_(3, 16) matrix columns and the reading frame in the *S* sequence coincide. In Fig. 5, this area is designated as *V*1. The second part (Fig. 5, *V*2) accounts for coincidences of the following form: 1 => 2, 2 => 3, 3 => 1, and the third part (Fig. 5, *V*3) falls on coincidences of the following form: 1 => 3, 2 => 1, 3 => 2. The sum of *V*1 + *V*2 + *V*3 − *kd* is equal to *mF*_max_, where *k* is the number of inserts or deletions, and *d* is the price for insertion or deletion from Equation (2). Initially, the *W*_1_(3, 16) matrix is unconnected with the reading frame. Therefore, cyclic rearrangements of the *W*_1_(3, 16) matrix were carried out, so that *V*1 *≥ V*2 and *V*1 *≥ V*3. As an indicator that could tell us about the presence of phase shifts of TP, we assumed *V*2 + *V*3.

For each gene we defined a threshold *V*0 = *V*2 + *V*3, below which it could be said that there were no TP phase shifts in the sequence. The *V*0 value was selected for each *S* sequence, using the *SR* sequences set. As gene sequences possess various lengths and different *MT* matrixes,^29^ the selection has to be done for each gene. The *SR* set contained 10^3^*S* sequences, where the codons were randomly shuffled. Such a shuffling obviously destroys all probable TP phase shifts. We selected the *V*0 value that provided no more than 20 sequences with random insertions or deletions in the *SR* set. Such insertions or deletions were considered to be significant [*N*(*V*0) ≤ 20], where *N* is the number of sequences with a random shift of TP. If *V*2 + *V*3 = 0, then the *SR* set was not created, because there were no TP phase shifts in the sequence.

## 3. Results and discussion

### 3.1. Estimation of the first and second type error rates of the developed method

In order to determine the number of errors of the first and the second type, we used all the coding sequences in the *A. thaliana* genome, which constitutes 48,322 cds. These sequences were downloaded from the Ensembl website (ftp://ftp.ensemblgenomes.org/pub/release-38/plants/fasta/arabidopsis_thaliana/cds/). We replaced all symbols (except *a*, *t*, *c* and *g*) in these sequences with a randomly selected nucleotide. Such a replacement was made for all studied cds.

Then, the *RN* set was created by the random shuffling of codons of the sequences from the initial set. Sequences from the random set should not contain TP phase shifts while having the same statistical properties as sequences from the initial set. The *RN* set allows estimation of the number of errors of the first type (true positive). We applied the approach developed by us to sequences from the *RN* set. In the result, we found 1,098 sequences with the TP phase shift, with *V*2 + *V*3 value higher than the threshold level. The total number of TP phase shifts in these sequences was 1,549.

It is also interesting to determine the number of errors of the second type and the power of the method. To do this, another test set (*RD*) was created using coding sequences from the *A. thaliana* genome having a length longer than 500 nt (40,621 sequences). The codons in these sequences were randomly shuffled. Then, a single-base deletion was introduced in a random position of each sequence not closer than 100 nt from the beginning or the end of the sequence. These sequences were analysed using the developed algorithm, and the results are presented in Table 2. This table shows that the method identified 28,357 sequences in the *RD* set, where *V*2 + *V*3 ≥ *V*0, and the predicted position is in the range of ±50 from the artificial deletion. This shows that second type errors constitute 30%, and so the power of the method constitutes 70%. Also, the method rather accurately predicts the location of the frameshifts, because it was only in 1,128 sequences that frameshifts were found outside the region ±50 from the deletion point. It should also be noted that the method does not create a significant number of random frameshifts. This is because 29,485 sequences with statistically significant frameshifts (28,357 + 1,128) contain 29,888 shifts, i.e. 403 frameshifts are due to purely random factors.

**Table 2:** Search for phase shifts in the set RD

Name of organism	The total number of sequences in the *RD* set	Number of sequences which have *V*2 + *V*3 ≠ 0	Number of sequences which have *V*2 + *V*3 ≥ *V*0		Total number of shifts
			Within ±50	Out ± 50	
1. *Arabidopsis thaliana*	40,612	31,203	28,357	1,128	29,888
2. *Anaeromyxobacter dehalogenans*	3,460	3,458	2,975	68	3,668

### 3.2. Searching for potential frameshift mutations in coding sequences from the *Arabidopsis thaliana* genome and several other eukaryotic genomes

All coding sequences were analysed from the *A. thaliana* genome. In the result, we identified 9,930 cds with one or more TP phase shifts, which could indicate the presence of frameshift mutations in these sequences. A total of 14,951 TP phase shifts were found in these cds. This indicates that in many cds we detected multiple TP phase shifts. As the number of false positives within the *RN* set constitutes 1,549 TP shifts (see Section 3.1), then the first type error rate could be estimated as about ∼11%. As we are dealing with cds derived from mRNA, we also excluded the TP phase shifts found in the cds obtained by the alternative splicing of the same gene. In this case, 6,624 unique cds remain in the *A. thaliana* genome.

For further analysis, the sequences where the TP phase shifts were found were divided into subsequences in accordance with the TP phase shift coordinates. Therefore, each sequence was split at least into two subsequences. Further, subsequences longer than 60 nt were translated into amino acid sequences in accordance with two frames (except the one, which was already present in the original gene). Two alternative frames must be considered, because we do not know which of the subsequences possesses the correct frame. If we have a single TP phase shift in the *x* coordinate, the reading frameshift could be registered in the sequence from 1 to *x* or in the sequence from *x* to the end of cds. The developed method is incapable of distinguishing between these two cases, and we cannot exclude the possibility that there was a frameshift at the beginning of the gene. As a result, 43,499 sequences longer than 20 amino acids were obtained. Next, for these sequences, the blastP program was employed to search against the Swiss-Prot database (*E*-value cut-off 0.1). Consequently, a similarity was found for 824 subsequences from the 774 cds. This means that for ∼774 cds, the frameshift was also confirmed by the amino acid sequence similarity.

Let us consider an example of a cds with a TP phase shift from the *A. thaliana* genome, for which a similarity was found using an alternative reading frame. The sequence identifier is AT1G79920.2, and the corresponding amino acid sequence identifier in the Swiss-Prot is F4HQD5_ARATH. A TP phase shift was found at the 1933 position, and thereafter, the frame changes from the first to the third. Table 3 presents the resulting $W_{2}^{t}(3,16)$ matrix. For the third reading frame, an amino acid sequence was also obtained. Table 4 shows that the F4HQD5_ARATH sequence possesses a similarity to the HS105_CRIGR sequence from the *Cricetulus griseus* (Chinese hamster) genome only after 642 amino acids, which corresponds to the coordinate of the discovered frameshift. The *E*-value for the similarity found constitutes 4.6e^−144^. Beyond the 656 amino acids of the HS105_CRIGR sequence, its similarity was observed with the amino acid sequence created by the third reading frame, after the 1933 position from the AT1G79920.2 cds (Table 5). The *E*-value for this similarity constitutes 4.6e^−19^. This example obviously demonstrates that simultaneously there are two similar proteins, one of which possesses a reading frameshift, while the other does not. The first one is the heat shock protein 70 (F4HQD5_ARATH) from the *A. thaliana* genome, while the second one is the heat shock protein 105 kDa (HS105_CRIGR) from the *Cricetulus griseus* (Chinese hamster) genome.

**Table 3:** The matrix $W_{2}^{t}(a(i),n)$ (see Equation 2) that was used for the construction of the final global alignment of cds AT1G79920.2 (point 2.4)

		A	T	C	G
1	A	−1.4	1.6	0.3	5.3
1	T	−7.9	3.5	2.4	1.7
1	C	−0.8	7.1	−0.5	−3.4
1	G	−6.3	−2.0	−1.6	−4.5
2	A	−3.5	−4.6	−4.7	0.9
2	T	−2.4	−1.9	0.7	11.3
2	C	1.3	−0.3	−0.3	−1.4
2	G	0.1	−3.7	−0.3	1.8
3	A	2.7	0.4	−0.3	−2.9
3	T	−6.8	0.2	−0.3	−5.4
3	C	−1.7	−1.1	0.4	−3.7
3	G	6.4	1.5	4.3	−0.9

*n *=* s*(*k*)+4(*s*(*i*) − 1), and *a*(*i*) was calculated as *i* – 3int(*i*/3) for *i *=* *1,…, *N.* The index *k* is calculated using the already created transitions in the matrix *F* (see the text under Equation 2). Columns 3 through 6 correspond to the bases *s*(*i*), the second column corresponds to the bases *s*(*k*), and the first column shows the positions *a*(*i*).

**Table 4:** Alignment of the amino acid sequence F4HQD5_ARATH, which is encoded by cds AT1G79920.2 from the *Arabidopsis thaliana* genome with the amino acid sequence HS105_CRIGR from the genome *Cricetulus griseus* (Chinese hamster)

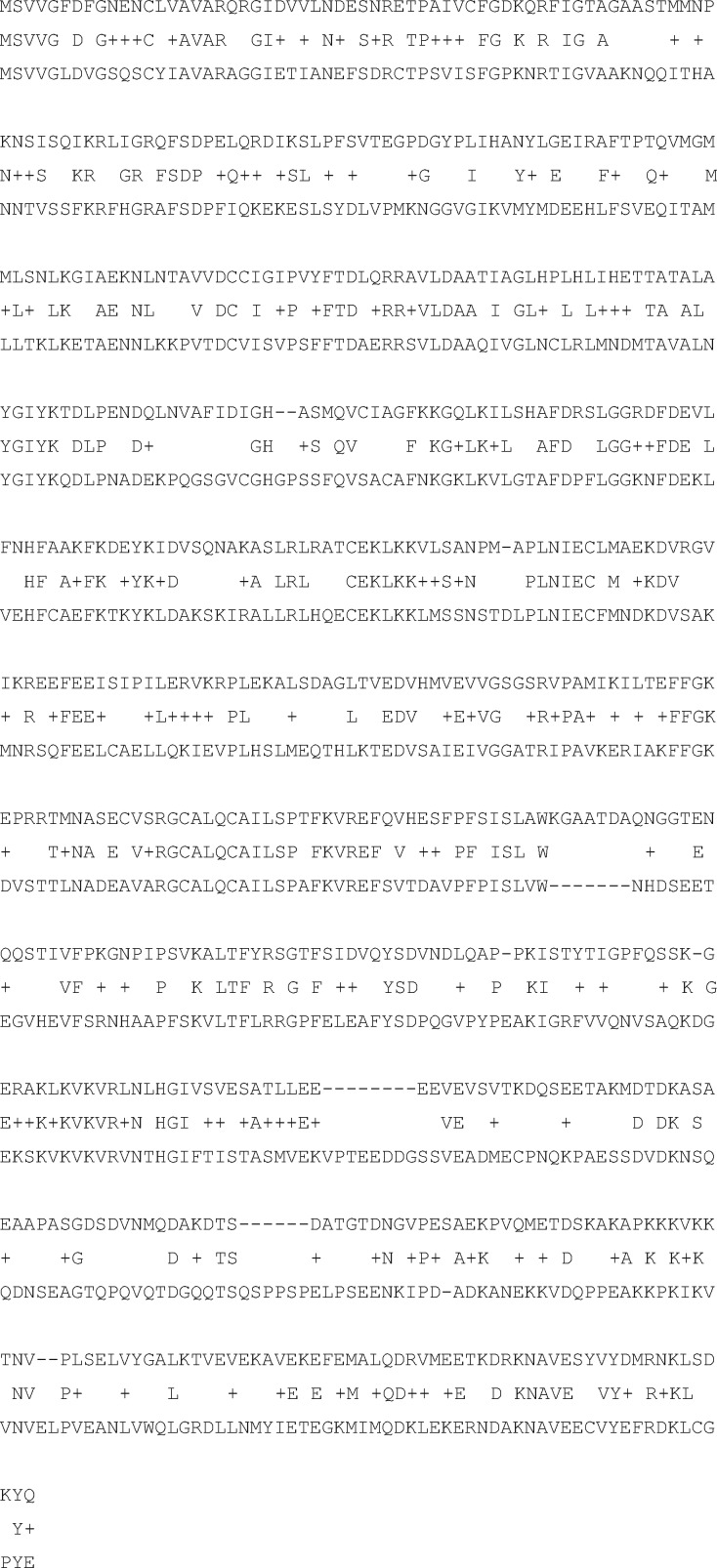

This alignment can be found from 1 to 642 amino acids for F4HQD5_ARATH and from 1 to 655 amino acids for HS105_CRIGR.

**Table 5:** Alignment of the amino acid sequence obtained by the third reading frame of the cds AT1G79920.2 from the position 1933 to the end of the sequence from the *Arabidopsis thaliana* genome, with the amino acid sequence HS105_CRIGR from the genome *Cricetulus griseus* (Chinese hamster)

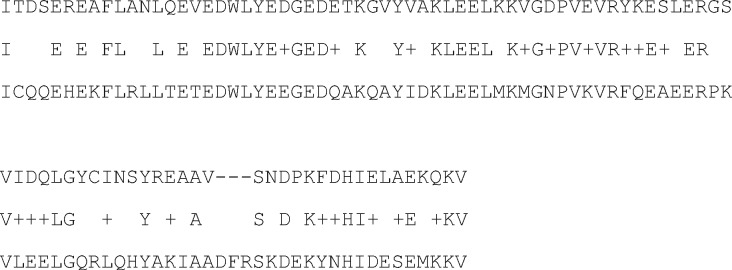

This alignment was found from 656 to 752 amino acids for the sequence HS105_CRIGR.

In addition to the *A. thaliana* genome, we applied our method to cds from five eukaryotic genomes. The cds were also obtained from the Ensembl database (ftp://ftp.ensembl.org/pub/release-91/fasta/). These genomes include those of *C. elegans, D. melanogaster, H. sapiens, R. norvegicus* and *X. tropicalis*. Table 6 presents data on the number of potential reading frameshifts. The level of errors of the first and second type corresponds to those estimated for the *A. thaliana* genome, with accuracy of ±5%. From Table 6, it can be seen that these genomes contain on average from ∼1.5 to 3 frameshifts in one *cds.*

**Table 6:** The number of cds with potential frameshift mutations in the six eukaryotic genomes examined, disregarding and taking into account alternative splicing

Organism name	Number of potential frameshift mutations	Number of cds with the potential frameshift mutations	Number of cds with potential frameshift mutations take into account an alternative splicing	Number of cds with potential frameshift mutations from the work^21^
*Arabidopsis thaliana*	14,954	9,930	6,624	2,067
Caenorhabditis *elegans*	11,103	6,370	3,788	611
Drosophila *melanogaster*	31,873	8,833	3,649	2,616
*Homo sapiens*	33,336	21,363	9,456	7,395
Rattus *norvegicus*	9,752	5,689	4,608	703
*Xenopus tropicalis*	6,348	4,014	3,401	529

The data obtained in the work^37^ are also shown.

### 3.3. Searching for potential frameshift mutations in coding sequences from prokaryotic genomes

We also studied the presence of TP phase shifts in cds from 17 bacterial genomes. For example, using the *A. deehalogenans* genome, we studied the *RN* and *RD* sets (see Section 3.1) created from the cds set of this genome. The results of examining the *RN* set (codon-shuffled sequences) demonstrate that only 61 TP shifts could be identified. Thus, the number of errors of the first type (false positives) for this genome is about 8% of the number of cds found with the TP phase shift (Table 7, Column 3). In the remaining 16 genomes, fluctuations in first type error rates ranged from 6% to 14%.

**Table 7:** The number of potential frameshifts in cds from the 17 bacterial genomes (Column 2) obtained in the present study

	This work		GeneTack-GM program			
1. Name of bacteria	2. Number of cds with frameshifts	3. Number of frameshifts	4. Inside the gene, no closer than 50 nt to the border	5. The beginning or end of the gene is not more than 50 nt (the number of cases with the addition of a neighbouring gene)	6. Between genes	7. Total
*Anaeromyxobacter dehalogenans*	425	768	49	101 (62)	32	182
*Archaeoglobus fulgidus*	77	174	44	299 (251)	53	396
*Bacillus subtilis*	126	434	35	79 (57)	27	141
*Campylobacter jejuni*	48	86	8	104 (92)	16	128
*Caulobacter crescentus*	306	651	43	57 (30)	3	103
*Clavibacter michiganensis*	357	736	35	65 (40)	63	163
*Methanopyrus kandleri*	150	436	76	96 (68)	38	210
*Methanosphaera stadtmanae*	111	272	5	37 (27)	13	55
*Pasteurella multocida*	46	146	9	56 (48)	4	69
*Picrophilus torridus*	15	62	7	154 (130)	9	170
*Pyrobaculum aerophilum*	71	249	65	235 (163)	48	348
*Ralstonia solanacearum*	330	607	38	66 (41)	19	123
*Salmonella enterica,*	115	457	52	162 (112)	38	252
*Staphylococcus aureus*	130	381	4	52 (43)	16	72
*Streptococcus pyogenes*	60	209	17	52 (39)	6	75
*Thermococcus kodakarensis*	85	172	43	57 (38)	4	104
*Thermotoga maritima*	37	127	10	76 (64)	5	91

Columns 4–7 show the data obtained in the work.^37^ Column 3 shows the shifts found within known genes (no closer than 50 nt before the start/end of the sequence). Column 4 shows the shifts that occur on the edges of known genes (no more than 50 nt from the beginning or end of a known gene). In parentheses, the number of shifts is indicated, when the association with the adjacent gene occurs. The number of shifts occurring in the area between known genes is shown in Column 5.

Table 2 presents the results obtained from studying the *RD* set. This table shows that from 3,460 codon-shuffled cds with an artificial frameshift, in 2,975 sequences, TP phase shifts were found within the ±50 nt interval. In 68 cases, TP phase shifts were predicted outside the ±50 nt region from the artificially created deletion. This implies that the number of errors of the second type constitutes ∼14%, and the power of the method is ∼86%. The results of analysis of the TP phase shifts in the remaining 16 genomes are presented in Table 7, Column 2. The total number of TP phase shifts is shown in Table 7, Column 3. The table shows that the number of TP phase shifts in bacterial genes ranges from several dozens to hundreds per single genome.

### 3.4. Comparison with the Genetack-GM program

It would be interesting to compare the obtained results with the results obtained previously while searching for reading frameshifts. It has been recorded that the Genetack-GM program showed the best results among other frameshift prediction methods, hence our results were compared with this program.^17^ For this purpose, the complete sequences of the 17 prokaryotic genomes were downloaded from the Ensembl database^39^ and submitted to the GeneTack-GM software program. GeneTack-GM is a combination of the GeneMark program designed to indicate coding sequences in a genome, and the Genetack designed to search for potential frameshifts.^37^ In the case of prokaryotic sequences, the Genetack software program searches for cases of potential frameshifts that resulted in the splitting of a single coding sequence into two independent ones (the author claimed that, in modern databases they are usually represented by two separate genes). The result of the GeneTack-GM software is the predicted coordinates of a gene (usually a hypothetical gene) and the coordinate of a frameshift within the gene.

We compared the coordinates of frameshifts obtained by Genetack for the 17 bacterial genomes with the boundaries of known genes indicated in the annotation to the corresponding genomes in the Ensembl database. Frameshifts found by the GeneTack-GM were divided into three categories according to their position in the known genes. The results are presented in Table 7. The first category includes frameshifts found within the known genes (not closer than 50 nt to the start/end of the gene) (Table 7, Column 4). The second category includes frameshifts that occur at the edges of the known genes (not more than 50 nt from the start/end of the gene). For this category, it is also indicated in parentheses whether this frameshift leads to uniting a gene with the neighbouring one (i.e. to predicting a hypothetical gene), if the coordinate of the end of a hypothetical gene predicted by the software program captures the following gene by more than 100 nt (Table 7, Column 5). The third category includes frameshifts occurring in the area between the known genes (Table 7, Column 6). It is evident that most of the frameshifts found by GeneTack are pertaining to the second and third categories. This implies that the presence of these frameshifts is connected to the fact that the two adjacent genes that are indicated in the Ensembl database are combined by the GeneTack-GM software program into a single gene or into one gene that captures parts of these genes.

However, this study evaluated the presence of reading frameshifts in known genes (cds). At a distance no closer than 50 nt from the boundaries, our method found more than 70% of the frameshifts. This 70% relate to the first category of frameshifts (70% of the data in Table 7, Column 3). A comparison of 70% of Columns 3 and 4 shows that our approach found a significantly large number of reading frameshifts within the already known genes, compared with the GeneTack-GM software program, with a lower false positive rate (8–14% for our program versus 32% for the GeneTack software program).

Similar results were obtained when comparing the results obtained in the present study for the *A. thaliana* genome with the data presented in the GeneTack database for the genome. A total of 2,067 potential reading frameshifts were found in the *A. thaliana* genome by the authors of GeneTack, whereas we were able to detect 14,951 TP shift cases (see Table 6). It should be noted that we analysed only the cds, whereas the GeneTack database contains data for mRNA sequences, which also includes the non-coding sequences (5′ and 3′ untranslated regions). Therefore, we also divided the 2,067 reading frameshifts into three groups, as shown in Table 7. The first group includes frameshifts which are located inside the cds not closer than 50 nt from the beginning and end of the coding section. The second group includes frameshifts that are located at a distance not more than 50 nt from the ends of cds, and in the third group the frameshift corresponds to the mRNA non-coding regions. The first group includes 485, the second group includes 710, and the third group includes 872 reading frameshifts.

**Figure 6:**
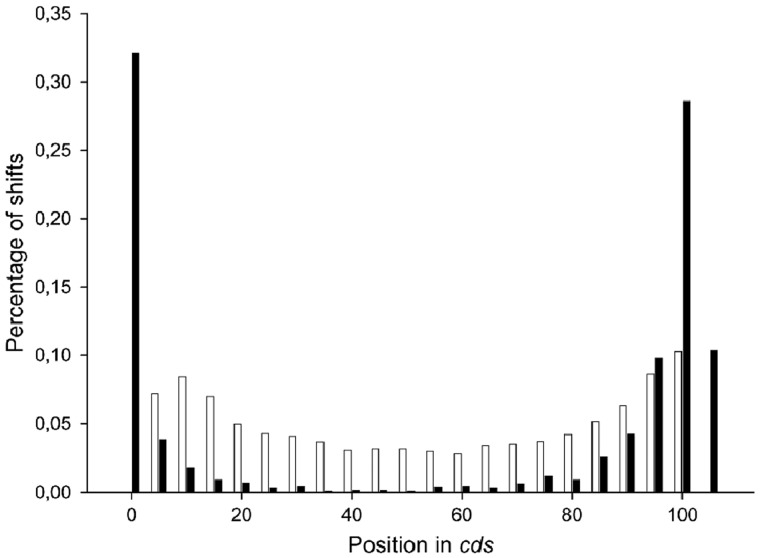
Distribution of shifts position in the sequence of a gene. The *x*-axis shows the distance as a percentage of the beginning of the gene (with step equals to 5%), the *y*-axis shows the percentage of shifts per interval of 5%. The black bars—the data from the work,^21^ the white—the data of our work. The leftmost and rightmost bars show the number of frameshifts found outside the cds from the work.^21^

**Figure 7:**
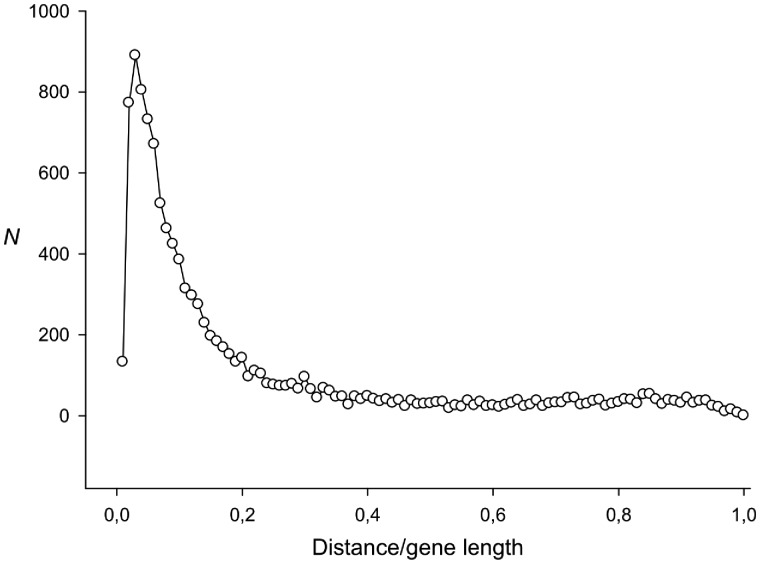
Distribution of the distance between paired compensating shifts of the TP phase in the *Arabidopsis thaliana* genome.

A more detailed study of the distribution of frameshifts by position in genes from the *A. thaliana* genome is shown in Fig. 6. The increase in the number of frameshifts at the end of cds may be due to the fact that the sequences at the end of the gene do not greatly affect the structure of the encoded protein. However, it is surprising that such an increase is also found at the beginning of the gene. It is difficult to imagine that such mutations will not change the biological function of the encoded protein. Rather, it can be assumed that the observed frameshifts are compensating, which return the reading frame to its original position. The initial frameshift could be at the very beginning of the gene and we were unable to see it using this method. Our approach may not find a frameshift due to a large penalty for insertion or deletion (*d* in Equation 2) if it occurs at a distance <20–30 bases from the start of the gene. However, the second, already found frameshift, just compensates it. In this case, the distance to the frameshift revealed at the beginning of the gene should be similar to the distance between the pairwise compensating frameshifts that we find in cds. We constructed a distribution between the pair compensating frameshifts. This distribution is shown in Fig. 7. It can be seen from the figure that the average distance between the compensating frameshifts is <0.1 of the corresponding gene size. This result supports our hypothesis that paired compensating frameshifts often occur at the beginning of a gene. This hypothesis explains the surprisingly large number of TP phase shifts, which was revealed at the beginning of the genes in Fig. 6.

As we have identified 14,951 potential reading frameshifts in cds from the genome, our algorithm is about seven times more efficient than the GeneTack-GM software program. If we compare the results in relation to the first group, then this difference will be greater, because more than 70% of the frameshifts detected are related to the first group (Fig. 6). Similar results were also obtained for all other eukaryotic genomes (Table 6). The result could be explained by the fact that if the HMM is trained on a set of selected mRNAs,^21^ statistical properties, such as *k*-mer frequencies, are averaged over the set. In the result of the averaging, the HMM parameters are changed so that the number of errors of the first and the second type could be increased. Consequently, some frameshifts could be missed by the HMM-based method. Let us illustrate this statement for brevity and simplicity using the classical Markov models. Consider two sequences, *seq*1 = ‘*ttgccagagcagattgcccagattgccagatt*’ and *seq*2 = ‘*aactcggtaacggtctaaactcggtacggtcta*’. The conditional probabilities of the nucleotide pairs of these sequences are presented in *N*1 and *N*2 (Table 8A and B). The matrix represents the probability *P*(*X_n_*_+1_ = *i_n_*_+1_|*X_n_* = *i _n_*), where *X_n_*_+1_ corresponds to the rows of the matrix, and *X_n_* corresponds to the columns. A Markov model was built using the conditional probability matrix *N*1 or the *N*2. The matrices *N*1 and *N*2 can be used to search for sequences with similar nucleotide correlations as the sequences *seq*1 and *seq*2. Let the probabilities *P*11 and *P*22 be the probabilities that the sequences *seq*1 and *seq*2 are generated by the *N*1 and *N*2 matrices, respectively [*P*11 = (0.5)^22^ and *P*22 = (0.5)^22^]. The probability *P*12 that the sequence *seq*1 was generated using the matrix *N*2 is equal to zero [*P*12 = (0)^22^ = 0] as well as the probability *P*21 (that the sequence *seq*2 was generated using the matrix *N*1). However, the probability that a randomly shuffled sequence was generated using the matrix *N*1 *PR*1 = 0 is zero, *PR*2 = 0 because in the randomly shuffled sequence there will be pairs of nucleotides, for which *P*(*X_n_*_+1_ = *i _n_*_+1_|*X_n_* = *i_n_*) = 0 both in the matrix *N*1 and in the matrix *N*2. Therefore, one can find sequences similar to *seq*1 or *seq*2 surrounded by random sequences using the Markov model at a statistically significant level.

**Table 8:** The matrices of conditional probabilities *P*(*X_n_*_+1_=*i _n_*_+1_|*X_n_*=*i _n_*), created by the sequences *seq*1 = ttgccagagcagattgcccagattgccagatt (A), *seq*2 = aactcggtaacggtctaaactcggtacggtcta (B) and unification of these sequences (C)

(A)							(B)					(C)			
	a	t	c	g			a	t	c	g		a	t	c	g
a	0	0.5	0	0.5		a	0.5	0	0.5	0	a	0.25	0.25	0.25	0.25
t	0	0.5	0	0.5		t	0.5	0	0.5	0	t	0.25	0.25	0.25	0.25
c	0.5	0	0.5	0		c	0	0.5	0	0.5	c	0.25	0.25	0.25	0.25
g	0.5	0	0.5	0		g	0	0.5	0	0.5	g	0.25	0.25	0.25	0.25

*X_n_*_+1_ corresponds to the rows of the matrix, and *X_n_* corresponds to the columns of the matrix.

But if a Markov model is trained using both sequences *seq*1 and *seq*2, the matrix *N*3 would be constructed (Table 8C). In this case, the probabilities of generating *seq*1 and *seq*2 are (0.25)^22^ and the same probability will be obtained for any other sequence of the same length, including the randomly shuffled sequence. So, the identification of *seq*1 and *seq*2 using the Markov model is impossible at a statistically significant level. We can say that the statistical properties of these sequences are averaged.

The same phenomenon can be observed in the case of real genes, when a training sample is created for HMM from many genes. This effect was employed on the Genetack-GM program. We created two sets of artificial genes (*Q*1 and *Q*2) with different types of triplet frequency using different synonymous codons and used the sets to train HMM. Each artificial gene had a length of 1,500 bases and contained a start codon as well as a stop codon. Each set has a volume of 1,000 sequences. Also, we created a set *Q*3 of 1,000 sequences, half of which were of type *Q*1, while the other half were of type *Q*2. Additionally we created two sets *D*1 and *D*2, which had the same TP as the sets *Q*1 and *Q*2, respectively. But each sequence contained one deletion in a random position, but not closer than 100 bases from the beginning or the end. In order to exclude the effect of the stop codons resulting from the frameshifts, we replaced them with randomly selected coding codons. The volume of sets *D*1 and *D*2 was 100 sequences each.

First, we trained the Genetack-GM program on sets *Q*1 and *Q*2 and searched for frameshifts in sets *D*1 and *D*2, respectively. The program found shifts in 75 sequences from set *D*1 and 17 from set *D*2. Trained *Q*3 Genetack-GM was applied to sets *D*1 or *D*2. In the result, the program found 8 and 0 sequences with frameshifts, respectively. This example shows that combining different genes into one training set can significantly degrade the capabilities of the HMM. In our method, such averaging does not occur because we analyse each cds without using a training set; the method is adjusted to the TP that exists in each considered cds. This means that the mathematical method finds an optimal correlation matrix considering the possibility of insertions or deletions for each analysed cds. The final alignment of the studied sequence against the obtained matrix provides an alignment and coordinates of the potential reading frameshifts. It is also important to note that to search for potential frameshifts using our method, one needs only cds without any other information or training set. This is the main improvement of the method in comparison with HMM to determine the reading frameshifts.

Genetack performance was also tested on a set of cds without additional non-coding regions. We randomly selected 1,000 cds from the *A. thaliana* genome. In each of these cds, we deleted a single nucleotide in a random position (but no closer than 100 nt from the beginning or end of the sequence). Then, we inserted a random nucleotide just before the stop-codon to keep the sequence length. Genetack and the method developed here were applied to this set. In the result, Genetack predicted frameshift in 615 sequences and our method in 676 sequences (inside ± 50 nt from the actual deletion position). The HMM for Genetack was trained by GeneMark on a set of 100,000 cds from *A. thaliana.* Then in each cds with deletion, we changed the stop and start codons that occurred after the deletion in the first frame to a randomly chosen coding codon. Then, we again applied both programs to this set. In the result, Genetack correctly predicts frameshift only in 277 sequences and our method in 624. The reduced number of frameshifts found by our method could be explained by the decreasing TP quality after the codon changes. The result demonstrates that Genetack is more suitable for the detection of frameshift that separates a single coding sequence into two (or more) independent genes as it was pointed by the authors.^17^ But Genetack weakly predicts frameshifts if they do not lead to the formation of the premature stop-codon or if we have only cds without surrounding non-coding regions.

We identified TP phase shifts in 660 genes, which constituted 83% of the first group, out of 1,183 genes discovered by the GeneTack software program in cds (2,080 genes found in cds and non-coding regions in the work^21^). Besides, more than 70% of the frameshifts found in our earlier publications for prokaryotic genes were identified using the developed algorithm.^30^^,^^40^ At the same time, for prokaryotic genes, a higher number of potential frameshift mutations was discovered using this method. This is because the developed method works much better with sequences having a low level of TP.

### 3.5. Discussion of the possibility of creating new genes through frameshift mutations

For many years, a study of the evolution of genes has attracted the attention of researchers. After determining the sequences of many prokaryotic and eukaryotic genes, the research in this area has significantly expanded. It is now believed that new genes are created by duplicating already existing genes.^41^ Therefore, a large number of genes are grouped in families based on the similarity of the amino acid or nucleotide sequences.^42^ Processes such as gene fusion,^43^ exon shuffling,^44^ alternative splicing^45^ and lateral gene transfer^46^ are considered to be the principal mechanisms of the creation of a variety of genes in a genome and the corresponding proteins. However, using such mechanisms, it is difficult to create a fundamentally new sequence, but a frameshift mutation could do this efficiently. It has previously been suggested that frameshift mutations could play a significant role in the process of creating new genes.^8^^,^^9^ The authors of the presented works have proposed that if a protein for some reasons is not under the selection pressure, frameshift mutation could persist and eventually lead to functional divergence. It is mainly this phenomenon that we observed in the present work. More than 20% of the studied eukaryotic cds contain potential frameshift mutations. The question that remains is, how does a protein sequence bear any biological sense after the reading frameshift? It could be assumed that the genetic code could be perfectly adapted to such changes, and it allows the frameshift mutations to obtain biologically meaningful sequences.

Search for frameshifts in genes by the developed method can be done online at: http://victoria.biengi.ac.ru/fsfinder.
